# Diagnosis of extracranial carotid stenosis by MRA of the brain

**DOI:** 10.1038/s41598-021-91511-w

**Published:** 2021-06-08

**Authors:** Chia-Hung Wu, Shu-Ting Chen, Jung-Hsuan Chen, Chih-Ping Chung, Chao-Bao Luo, Wei-Hsin Yuan, Feng-Chi Chang, Han-Hwa Hu

**Affiliations:** 1 Department of Radiology, Taipei Veterans General Hospital, No. 201, Section 2, Shipai Road, Beitou District, Taipei, 11217 Taiwan; 2 Department of Neurology, Neurological Institute, Taipei Veterans General Hospital, Taipei, Taiwan; 3 School of Medicine, National Yang-Ming University, Taipei, Taiwan; 4 School of Medicine, National Yang Ming Chiao Tung University, Taipei, Taiwan; 5 Institute of Clinical Medicine, National Yang-Ming University, Taipei, Taiwan; 6 Institute of Clinical Medicine, National Yang Ming Chiao Tung University, Taipei, Taiwan; 7 Department of Biomedical Engineering, Yuanpei University of Medical Technology, Hsinchu, Taiwan; 8 Division of Radiology, Taipei Municipal Gan-Dau Hospital (Managed by Taipei Veterans General Hospital), Taipei, Taiwan

**Keywords:** Health care, Neurology, Signs and symptoms

## Abstract

Severe extracranial carotid stenosis (SECS) patients may present with nonspecific neurological symptoms that require intracranial magnetic resonance imaging (MRI) and time-of-flight (TOF)-MR angiography (MRA) to exclude intracranial pathology. Recognition of SECS on intracranial TOF-MRA findings is beneficial to provide a prompt carotid imaging study and aggressive stroke prevention. Patients with SECS (January 2016 to May 2019) undergoing percutaneous transluminal angioplasty and stenting (PTAS) were included. Differences in normalized signal intensities (SR_ICA_) and diameters (D_ICA_) between bilateral petrous internal carotid arteries (ICAs) were calculated 1 cm from the orifice. A hypothesized criterion describing the opacification grades (G_OPH_) of bilateral ophthalmic arteries was proposed. We correlated SR_ICA_ (*p* = 0.041), D_ICA_ (*p* = 0.001) and G_OPH_ (*p* = 0.012), with the severity of extracranial carotid stenosis on digital subtractive angiography (DSA) in the examined group (n = 113), and all showed statistical significance in predicting percentages of ICA stenosis. The results were further validated in another patient group with SECS after radiation therapy (n = 20; *p* = 0.704 between the actual and predicted stenosis grades). Our findings support the evaluation of the signal ratio and diameter of intracranial ICA on TOF-MRA to achieve early diagnosis and provide appropriate management of SECS.

## Introduction

Severe extracranial carotid stenosis (SECS) is one of the major causes of ischemic stroke^1,2^. Beside cerebral ischemic symptoms, SECS can present with various neurological symptoms, including dizziness, syncope, headache, involuntary limb movement, visual impairment and cognitive decline^3–5^. For these nonspecific neurological symptoms, evaluation of SECS by ultrasonography or time-of-flight-magnetic resonance angiography (TOF-MRA) of the neck is not regularly applied and may even been overlooked in some instances. Instead, MRI and TOF-MRA of the brain are usually done to confirm and/or exclude the diagnosis of tumors of cerebellopontine angle, cerebral aneurysm, seizure, brain tumors or dementia. Early diagnosis of SECS on TOF-MRA of the brain is important to provide the most appropriate treatment and thus reduce the risk of further ischemic symptoms^6^. Some studies have developed clinical prediction rules in patients with asymptomatic carotid stenosis^7–10^. However, the reported prediction usually requires complicated clinical and laboratory data. Using intracranial MRI to predict the possible existence of extracranial carotid stenosis is less often discussed^11–13^. Screening for patients with asymptomatic carotid stenosis is often performed by ultrasonography^14,15^, yet oversight exists due to the relatively low clinical awareness before the first ischemic symptoms^16^. To provide early diagnosis of SECS, this retrospective study was designed to evaluate the imaging characteristics of the intracranial TOF-MRA, including differences of signal intensities and diameters of bilateral petrous internal carotid arteries (ICAs) and opacification grades of ophthalmic artery in patients with unilateral SECS.

## Results

### Study subjects

The process of patient recruitment is summarized in Supplementary Fig. S1. Patients undergoing carotid DSA in our hospital from January 2016 to May 2019 were initially recruited (n = 181). After thorough inspection of the images, those with bilateral carotid stenosis (n = 24), suboptimal imaging (n = 5), tandem lesions in carotid arteries (n = 12) or previous intervention before the DSA (n = 7) were dropped from the study. In total, 133 patients were then separated into two groups. To examine the feasibility of the hypothesis in other patients with carotid stenosis by different etiologies, those with previous radiation therapy were enrolled in the validation group (n = 20), while others (n = 113) were in the examined group. The detailed demographics of both groups are displayed in Table 1.

**Table 1:** Demographics of the patients in the examined and validation groups.

	Examined Gr	Validation Gr	*p* values
Total patient number	113	20	
Female gender	26 (23.0%)	2 (10.0%)	0.188
Age (mean ± SD)	71.0 ± 10.1	63.4 ± 12.9	0.003
Days between MRI and angiography (mean)	21.2	28.7	0.275
Disease side (R:L)	68: 45	15: 5	< 0.001
Stenosis (%)	81.2 ± 10.2	79.5 ± 9.6	0.508

The distribution of the percentages of carotid stenosis in both groups showed no significant differences (81.2 ± 10.2 for the examined group and 79.5 ± 9.6 for the validation group, *p* = 0.508) (Table 1 and Supplementary Fig. S2).

### The examined group

The linear regression coefficients of the three variables, “G_OPH_”, “D_ICA_” and “SR_ICA_”, were 2.794, 52.056 and 15.765, respectively. (Table 2). The *p* values were 0.012 for “G_OPH_”, 0.001 for “D_ICA_”, and 0.041 for “SR_ICA_”. The variance inflation factors (VIFs) were 1.022 for “G_OPH_”, 1.237 for “D_ICA_”, and 1.232 for “SR_ICA_”.

**Table 2:** The coefficients of the linear regression of the three parameters derived from the examined group (n = 113, stenosis = 81.2 ± 10.2%).

	G_OPH_	D_ICA_	SR_ICA_
The linear regression coefficient	2.794	52.056	15.765
Significance (*p* value)	0.012	0.001	0.041
R square	0.076	0.184	0.123
Beta	0.212	0.320	0.189
Collinearity statistics (VIF)	1.022	1.237	1.232

*VIF *variance inflation factor.

### The validation group

The coefficients of the three variables generated in the examined group were further used to predict the possible stenosis percentages in the validation group. The *p* value regarding the differences between the predicted and actual stenosis percentages in the validation group was 0.704. The mean bias difference between the actual and predicted stenosis percentages was 0.845 and the mean absolute difference was 6.971 in percentage (Table 3).

**Table 3:** Actual stenosis and predicted stenosis in the validation group.

	**Actual stenosis**	**Predicted stenosis**
Stenosis (%)	79.5 ± 9.6	80.4 ± 6.0
Paired t test	p = 0.704	
Mean bias difference (%)	0.845	
Mean absolute difference (%)	6.971	

### The receiver operating characteristic (ROC) curves

**Figure 1:**
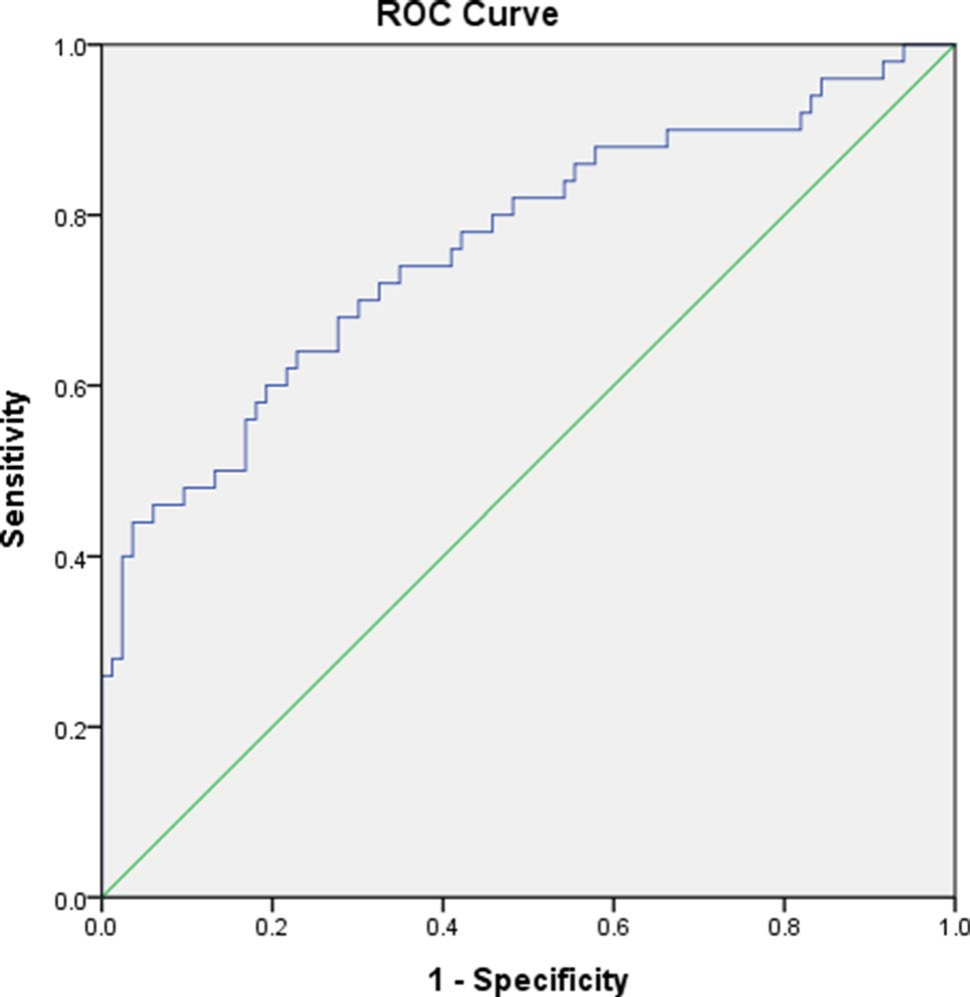
ROC curve (blue line) classifying the presence of 85% stenosis of the internal carotid arteries.

The areas under the ROC curve (AUC) after combining the three variables was 0.662 (Supplementary Fig. S3) regarding stenosis percentages less than 80% or not (95% confidence interval). The AUC was 0.763 when the threshold was set as 85% (Fig. 1).

### Interrater agreement

Single measures of the intraclass correlation coefficients were 0.973 for stenotic percentages on digital subtractive angiography (DSA), 0.877 for the variable “D_ICA_” and 0.952 for the variable “SR_ICA_”. The kappa value for “G_OPH_” was 0.749, which indicated substantial agreement^17^.

## Discussion

This study demonstrated that a routine TOF-MRA of the brain is able to predict SECS using the three imaging characteristics. These three imaging characteristics were all easily evaluated but might be overlooked. The performance of the evaluation tool was evaluated by the validation group and was not significantly different between the predicted and actual stenotic percentages (*p* = 0.704). To our knowledge, this is the largest human study to demonstrate the possible prediction of SECS by noncontrast intracranial TOF-MRA.

### Correlations of the three variables with stenotic percentages

The linear correlations of the three variables, “G_OPH_”, “D_ICA_” and “SR_ICA_”, were statistically significant. The *p* values of “G_OPH_” and “D_ICA_” were 0.012 and 0.001, respectively. The *p* value of “SR_ICA_” was relatively higher than the others (*p* = 0.041). Possible causes of the higher value, other than significance, were likely due to an additional normalization measure using basilar artery (BA) as the reference. The impact of the posterior circulation was an additional factor that was not included in the evaluation of the other two variables. Furthermore, we did not exclude patients with variations of Willis circle, including hypoplasia of the A1 segment of anterior cerebral artery, or the presence of fetal posterior cerebral artery, which may impair vascular flow^18^.

### Validation group

There was no significant difference between the percentages of actual stenosis (79.5 ± 9.6%) on DSA and the predicted stenosis (80.4 ± 6.0%) from the three variables, with correlation coefficients acquired from the examined group (*p* = 0.704) (detailed data in Supplementary Table S1). The study subjects in the validation group were those who had undergone radiation therapy, and the fitness of the prediction in this group proved that the imaging characteristics were mainly affected by the vascular flow instead of the etiology of the stenosis.

### The ROC curves

We evaluated the abilities of the three variables to classify the binary diagnosis, which was defined as those less than 80% stenosis or not on DSA. The reason for choosing 80% as the threshold was based on the indications of stenting in patients with asymptomatic carotid stenosis^19,20^. The AUC was 0.662, and it was 0.763 when the threshold of the stenotic percentage was set as 85%. The results demonstrated the higher classification ability of these variables in the binary diagnosis of patients with high-grade stenosis.

In this study, we aimed to investigate easily accessed parameters on TOF-MRA to warrant further surveys for possible extracranial carotid stenosis. The three parameters were clinically accessible and easily identified. The correlation of the ophthalmic artery and carotid flow on the transcranial Doppler (TCD) had been described in several studies^21,22^. The reversed flow of the ophthalmic arteries on TCD was an evident imaging characteristic in patients with severe carotid stenosis^21–23^. The reversed flow arose from the collaterals of the external carotid artery (ECA), usually but not limited to the middle meningeal artery, facial artery, superficial temporal artery and the maxillary artery^21^. Our study proved that a similar phenomenon could also be inspected on TOF-MRA as a loss of opacification. A possible cause of the diminished opacification of the ophthalmic arteries on TOF-MRA in patients with severe carotid stenosis was the concomitant stenosis of the ophthalmic arteries^23^. Both etiologies would result in the loss of forward flow on TCD and loss of opacification on TOF-MRA. The other two significant parameters on TOF-MRA measured the corrected signal intensities and opacified diameters of the petrous ICA. Decreased signal intensities and luminal diameters on TOF-MRA due to atherosclerosis or proximal vascular compromise had been described in other vascular diseases, including peripheral arterial disease^24,25^ and renal artery stenosis^26^. However, a similar imaging approach was less discussed in TOF-MRA of the brain. Considering the working mechanism of the TOF-MRA, severe stenosis or proximal compromise of the blood flow may result in decreased flow-related enhancement distal to the stenotic segments^27^. This mechanism was supportive of our hypothesis stating that the decreased signal intensity in the petrous ICA after normalization was the result of proximal vascular compromise. The similarity (*p* = 0.704) of the intracranial findings on TOF-MRA in patients with extracranial carotid stenosis with different etiologies (atherosclerosis in the examined group and radiation in the validation group) also supported this hypothesis that the signal or diameter change on TOF-MRA was a consequence of proximal vascular change^27^.

Other MR characteristics, including hypointense vessels on gradient echo^28^ and hyperintense ivy sign on T2-fluid attenuated inversion recovery (FLAIR)^29,30^, had been described in patients with compromised carotid vascular flow. However, these findings were usually found in advanced SECS, such as in the case of more than 90% carotid stenosis. They are also difficult to quantify. The use of the three parameters on TOF-MRA in this study offers sustainable and easily accessed parameters for possible extracranial carotid stenosis and is of clinical significance. This study also offers a future study possibility to set up a treatment response evaluation after percutaneous transluminal angioplasty and stenting (PTAS) of SECS. For long-term outcome follow-up of PTAS of SECS on TOF-MRA of the brain, these three parameters have the potential to diagnose significant restenosis.

### Strengths and limitations

There were several strengths in this study. First, this was the first and largest human study using intracranial TOF-MRA solely to predict possible high-grade carotid artery stenosis. Second, the findings on TOF-MRA were easily approached and evaluated and thus can be performed in real clinical settings. Third, the relatively high interrater agreement implies the easy accessibility and evaluation of the imaging characteristics.

One of the limitations was that the MR imaging was performed in different machines in our facility. To avoid the possible effects of smoothing or other postprocessing measures, we acquired the imaging characteristics on the source imaging of the TOF-MRA in each study subject. The heterogeneous nature of different MR machines creates possible imaging differences, yet the condition was more similar to the clinical reality. Another limitation was that the percentages of the carotid artery stenosis in both study groups (81.2 ± 10.2% in the examined group and 79.5 ± 9.6% in the validation group) were higher than the general population. This potential bias may limit the generalization, especially in subjects with less severe extracranial carotid stenosis. To avoid unnecessary radiation exposure from the DSA in patients who were not indicated for possible angioplasty or carotid stenting, we chose to only recruit study subjects who needed further DSA evaluation by MRI. Thus, this may have created certain selection bias. The unbalanced study design in the comparison between the examined group (n = 113) and the validation group (n = 20) may also lead to potential errors. Relatively small number of the patients after radiation in the validation group further created this difference. However, the major purpose of this article was not the precise prediction of stenotic percentages by intracranial TOF-MRA, which is unnecessary. The importance of this study was to characterize the easily identified red flag signs on TOF-MRA for clinical physicians to arrange further image surveys, including ultrasonography, to examine possible carotid artery stenosis.

Three imaging characteristics on TOF-MRA of the brain, including differences in the opacification grades of bilateral ophthalmic arteries and the diameters and signal intensities of bilateral petrous ICAs, were correlated strongly with the stenotic percentages in patients with SECS. We suggest evaluating these imaging findings of TOF-MRA of the brain in patients with risk factors of atherosclerosis. With the presence of these imaging characteristics, patients should undergo further carotid artery surveys.

## Methods

### Ethics

This study was approved by the Institutional Review Board of Taipei Veterans General hospital (code: 2019-07-026AC and 2019-07-002BC). All clinical investigations were conducted according to the principles expressed in the Declaration of Helsinki. The corresponding authors had full access to all data in the study and had final responsibility for the decision to submit the research for publication.

### Study design

This is a retrospective study. The data of the patients undergoing carotid DSA with unilateral SECS in Taipei Veterans General hospital from January 2016 to May 2019 was collected. Before further analysis, all the study subjects were informed of the image collection and signed the informed consent documents. The patients were further separated into two groups, the examined group and the validation group.

### Image analysis

**Figure 2:**
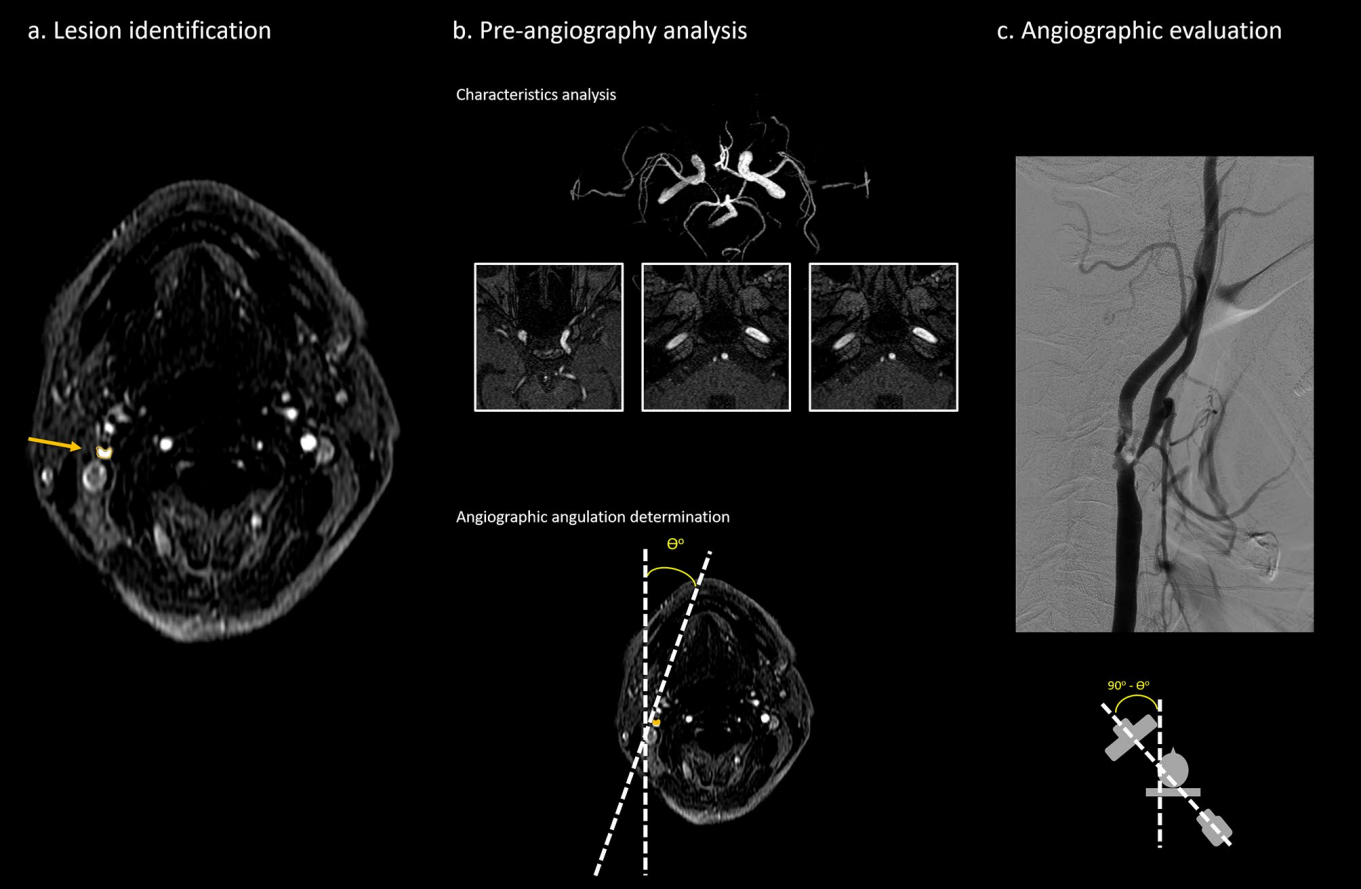
A standardized protocol in patients with potential carotid stenting indications. (**A**) Stenosis of the internal carotid artery (ICA) was identified on TOF-MRA. (**B**) The three imaging characteristics, including differences in the opacification grades of bilateral ophthalmic arteries and the signal intensities and diameters of bilateral petrous ICA, were evaluated. An angulation for further DSA was calculated to avoid discrepancy between operators. (**C**) DSA was performed based on the previously acquired angulation. The exact stenotic percentages were calculated based on the NASCET criteria.

MRI of the brain with time-of-flight (TOF)-MRA was performed before the DSA in each study subject. Carotid DSA was performed with a standardized protocol (Fig. 2). Angulation of the DSA gantry was planned according to the previous TOF-MRA to face the carotids at the stenotic level longitudinally to avoid the discrepancy between different operators.

MR images were acquired on a 3-Tesla (T) MRI machine (MR750, GE Healthcare, Milwaukee, WI, USA or Ingenia Elition, Koninklijke Philips N.V., Amsterdam, Netherlands). The TOF-MRA was performed in a 3 dimensional (3D)-manner (echo time/repetition time/slice thickness = 3.5 ms/24.0 ms/1.0 mm).

Two licensed neuroradiologists (C.H.W and F.C.C) conducted the imaging analysis. The images were read in a random order. The two readers evaluated the images separately and were blind to the randomized methods and patient information of each study subject. Final grading of the carotid stenosis, ophthalmic arteries opacification, and diameters and ratio of the signal intensities of the petrous ICA were based on the consensus of both readers when a discrepancy existed.

Stenosis grading was measured by the criteria in The North American Symptomatic Carotid Endarterectomy Trial (NASCET) on DSA^31^. Those with stenosis less than 30% were considered normal in this study.

**Figure 3:**
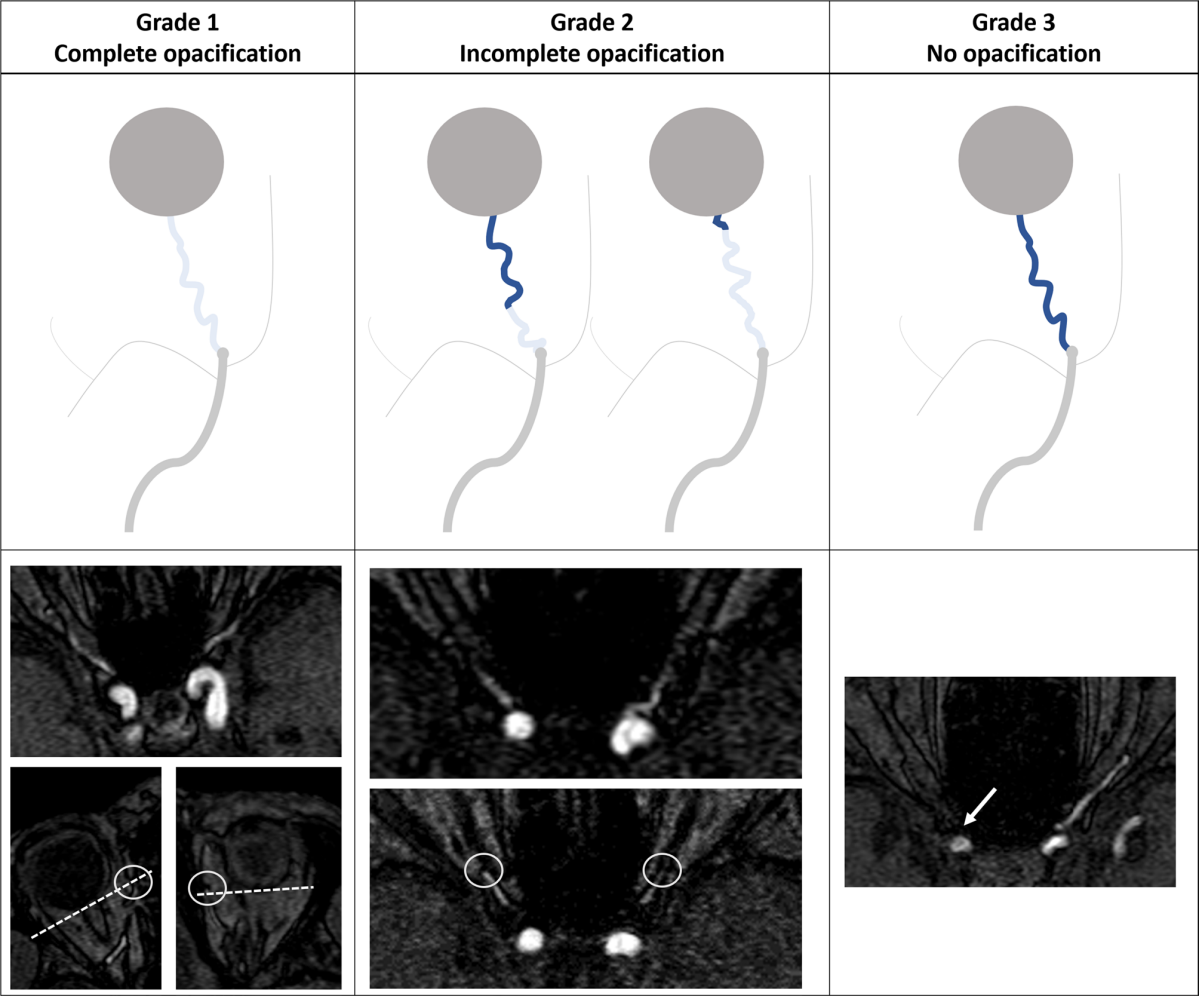
A proposed criterion of opacification grading in the ophthalmic artery. Those with visible distal opacification (circles) to the level of the posterior border of the globes (dotted lines), labeled as “complete opacification”, were grade 1. Those with complete loss of the visible ophthalmic opacification (arrow), labeled as “no opacification”, were grade 3. Any conditions other than grade 1 and 3 were labeled as grade 2, “incomplete opacification”.

Opacification of the ophthalmic artery was graded by a hypothesized criterion (Fig. 3). Those with visible distal opacification to the level of the posterior border of the globe, labeled as “complete opacification”, were grade 1. Those with complete loss of the visible ophthalmic opacification, labeled as “no opacification”, were grade 3. Any conditions other than grade 1 and 3 were labeled as grade 2, “incomplete opacification”.

**Figure 4:**
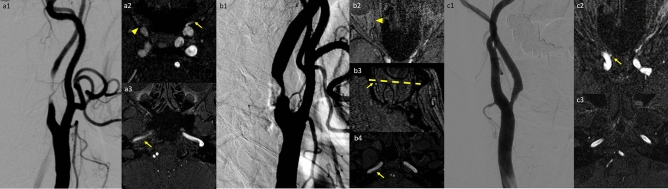
Demonstration of the three imaging parameters in three different patients with different stenosis percentages of the internal carotid arteries (ICAs). (**A**) Nearly total occlusion of the right ICA. Right grade 3 (arrowhead in **A2** for no visible opacification of the ophthalmic artery) and left grade 2 (arrow in **A2** for distal end of the ophthalmic artery opacification) of the ophthalmic artery opacification were depicted. Decreased diameter and signal intensities of the right petrous ICA (arrow in **A3**) compared to the contralateral side were noted. (**B**) Right ICA with 77.2% stenosis. Right grade 2 (arrowhead in **B2** for distal end of the ophthalmic artery opacification) and left grade 1 (arrow in **B3** for distal end of the ophthalmic artery opacification; the dotted line indicated the level of the posterior border of the globe) ophthalmic opacification were noted. Slightly decreased signal intensity without significant diameter discrepancy of the right petrous ICA (arrow in **B4**) compared to the contralateral side were demonstrated. (**C**) Left ICA with 59.4% stenosis. Bilateral grade 2 ophthalmic opacification with a tiny ophthalmic artery budding from the right ICA (arrow in **C2**). No significant discrepancy of signal intensities or diameter are depicted between bilateral petrous ICAs (**C3**).

The diameters of bilateral petrous segments of the internal carotid arteries were calculated. The signal intensities of bilateral petrous ICAs and the distal basilar artery were measured (Fig. 4). A fixed circular region of interest with 0.3 cm^2^ in area was applied 1 cm distal to the proximal end (orifice) of the bilateral petrous ICA on TOF-MRA. Another circular region of interest with 0.1 cm^2^ was placed onto the center of the distal tip of the basilar artery. To avoid the effect of signal loss due to the turbulent flow, the above ROIs were placed at the center of the vessels.

The above measurements of stenosis gratitude, the diameters and signal intensities of the vessels and the review of images were carried out on the picture archiving and communication system (PACS) developed in our hospital (SmartIris, Ver. 2.1.0.11, The Taiwan Electronic Data Processing Co., TEDPC, Taipei, Taiwan).

### Statistical analysis

Descriptive statistics are presented as the means ± standard deviation when applicable.

Three defined variables, “G_OPH_”, “D_ICA_” and “SR_ICA_”, were used in the statistical analysis. The “G_OPH_” was the result of each ophthalmic opacification grading on the diseased side minus the one on the healthy side. The variable “D_ICA_” was defined as the diameter of the petrous ICA on the healthy side minus the one on the diseased side. We defined the variable “SR_ICA_” as the results of the ratio of the difference in signal intensities between bilateral petrous ICAs to the signal intensities of the mid-basilar artery. The difference between bilateral petrous ICA was calculated as the healthy side minus the diseased side.

Linear regression was used to evaluate the possible correlation between the three variables and the actual stenosis gratitude measured on the DSA in the examined group. A collinear test examining variance inflation factors (VIF) was also performed to quantify the severity of multicollinearity. The results acquired from the examined group was further used to predict the possible stenosis in the validation group using the correlation between the three defined variables (“G_OPH_”, “D_ICA_” and “SR_ICA_”). The three variables calculated on the TOF-MRA in the validation group were applied to the linear regression coefficients acquired from the examined group (Table 2) to produce the predicted stenosis values in the validation group. The actual stenosis in the validation group was determined on DSA 28.7 ± 8.4 days after the TOF-MRA. Paired t test was carried out to evaluate the possible difference in the stenosis gratitude between the predicted and actual values in the validation group. The mean bias and absolute differences between the actual and predicted percentage stenosis values were also calculated.

Receiver operating characteristic (ROC) curves were generated using the combined three variables of patients in both groups regarding stenosis percentages less than 80% and 85% or not.

The interrater agreements were evaluated by single measures of the intraclass correlation coefficients (stenotic percentage of carotid arteries on DSA, “D_ICA_” and “SR_ICA_”)^32^ and Kappa values (“G_OPH_”)^17^.

All statistical analyses were performed with the Statistical Product and Service Solutions (SPSS, IBM Corporation, Armonk, NY) statistics software package, version 24.0.

## Supplementary Information

Supplementary Information.

## Data Availability

The datasets generated during and/or analyzed during the current study are available from the corresponding author on reasonable request.
