# Supplementary material for: Diagnosis of extracranial carotid stenosis by MRA of the brain
Source: Sci Rep. 2021 Jun 8;11:12010. doi: 10.1038/s41598-021-91511-w (PMC8187356; doi:10.1038/s41598-021-91511-w)

**Diagnosis of extracranial carotid stenosis by MRA of the brain**

Chia-Hung Wu, MD^1,3,4,5,6^, Shu-Ting Chen, MD^1,3,4^, Jung-Hsuan Chen^1,3,4^, Chih-Ping Chung MD, PhD^2,3,4^, Chao-Bao Luo, MD ^1,3,4,7^, Wei-Hsin Yuan, MD^1,3,4,8^, Feng-Chi Chang MD^1,3,4,*^, Han-Hwa Hu MD^2,3,4^

^1^Department of Radiology, Taipei Veterans General Hospital, Taipei, Taiwan

^2^Department of Neurology, Neurological Institute, Taipei Veterans General Hospital, Taipei, Taiwan

^3^School of Medicine, National Yang-Ming University, Taipei, Taiwan

^4^School of Medicine, National Yang Ming Chiao Tung University, Taipei, Taiwan

^5^Institute of Clinical Medicine, National Yang-Ming University, Taipei, Taiwan

^6^Institute of Clinical Medicine, National Yang Ming Chiao Tung University, Taipei, Taiwan

^7^Department of Biomedical Engineering, Yuanpei University of Medical Technology, Hsinchu, Taiwan

^8^Division of Radiology, Taipei Municipal Gan-Dau Hospital (managed by Taipei Veterans General Hospital), Taipei, Taiwan

*Corresponding author

**Supplementary Information**

**Supplementary Table S1** The complete data of the validation group.

**Supplementary Figure S1** Flow chart of the study subject recruitments.

**Supplementary Figure S2** Similar distributions of stenotic percentages of the internal carotid artery on DSA in the two groups.

**Supplementary Figure S3** ROC curve classifying the presence of 80% stenosis of the internal carotid arteries.

**Supplementary Table S1.** The complete data of the validation group.

| Actual stenosis (%) | Predicted stenosis (%) |
| --- | --- |
| 82.10 | 81.08 |
| 90.25 | 93.45 |
| 80.12 | 83.48 |
| 75.95 | 78.71 |
| 80.75 | 76.96 |
| 70.46 | 81.01 |
| 75.96 | 87.35 |
| 99.49 | 93.15 |
| 74.79 | 83.11 |
| 77.79 | 78.16 |
| 73.46 | 73.62 |
| 65.79 | 77.24 |
| 83.46 | 82.42 |
| 85.91 | 82.15 |
| 75.46 | 79.62 |
| 60.60 | 82.10 |
| 96.46 | 71.77 |
| 87.64 | 75.97 |
| 83.76 | 74.81 |
| 70.44 | 71.38 |

**Supplementary Figure S1.** Flow chart of the study subject recruitments.

*/***A true stenosis defined as those more than 30% stenosis on DSA.

**Suboptimal imaging includes those with motion artifacts, 2D only TOF-MRA, missing raw data of TOF-MRA, inadequate angulation of angiography or others

****Defined as previous stenting


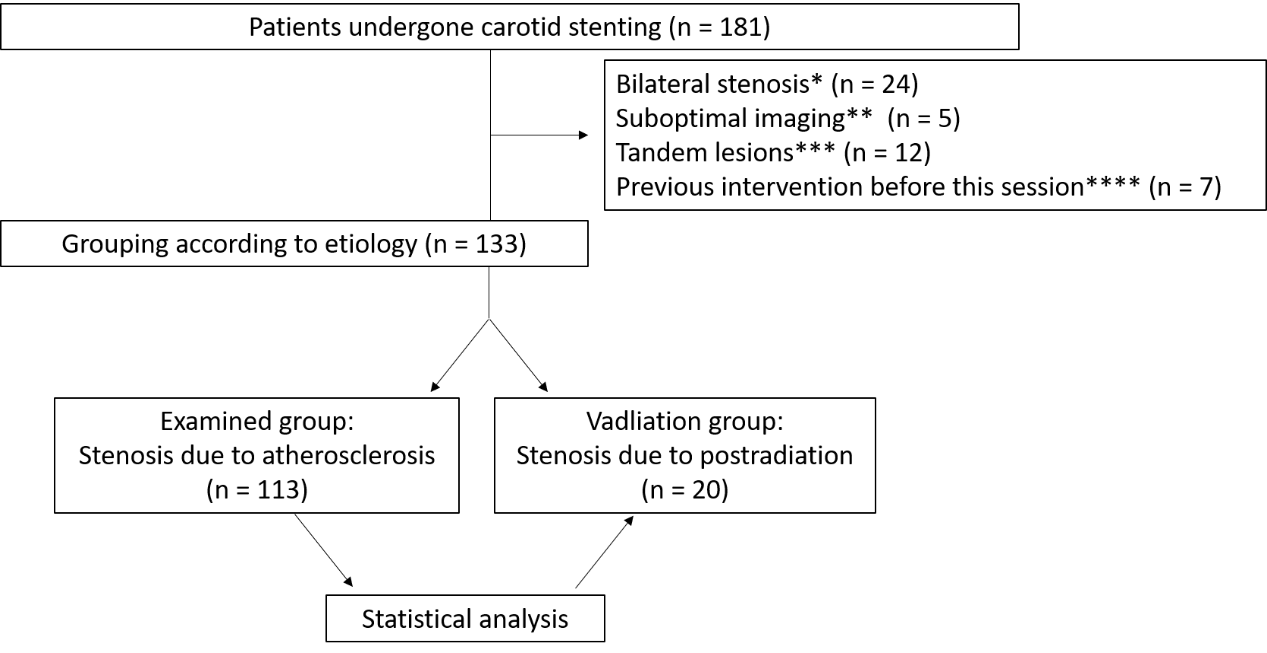


**Supplementary Figure S2.** Similar distributions of stenotic percentages of the internal carotid artery on DSA in the two groups.


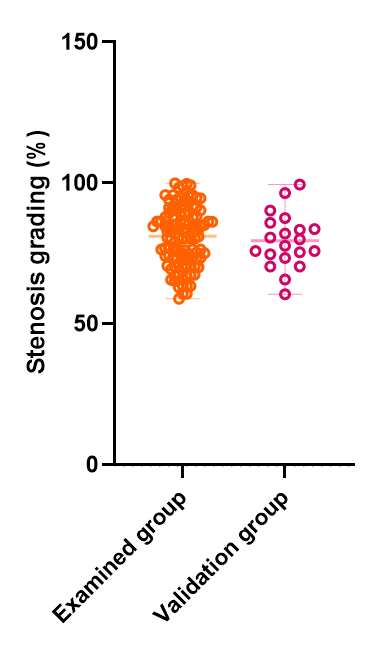


**Supplementary Figure S3.** ROC curve classifying the presence of 80% stenosis of the internal carotid arteries.


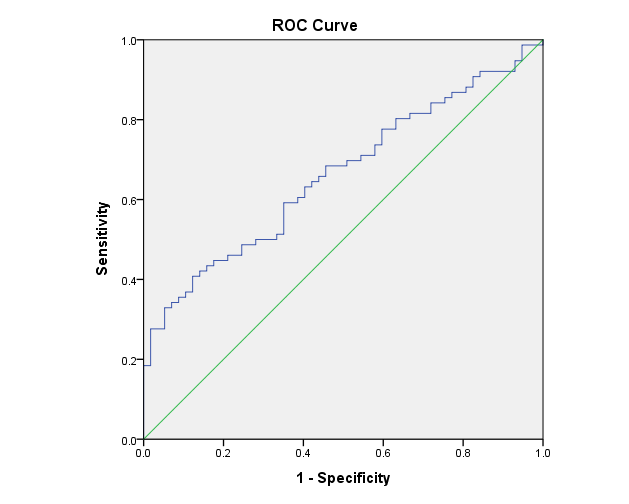

Supplement: Supplementary file 1 — Supplementary Information. [file 41598_2021_91511_MOESM1_ESM.docx]
